# Feasibility of Ultra-Dilute Iodine Contrast Agent in Dual-Layer Spectral CT for Pulmonary Artery Imaging

**DOI:** 10.2174/0115734056451922260515200533

**Published:** 2026-05-24

**Authors:** Yang Shan, Zhu Haifeng, Tian Yang, Wang Yujie, Wen Jing, Qin Jun

**Affiliations:** 1 Department of Radiology, Civil Aviation General Hospital, Beijing 100123, China; 2 CT Clinical Science, Philips Healthcare, Shenyang 110000, China

**Keywords:** X-ray computed tomography, Spectral computed tomography, Contrast media, Pulmonary artery imaging, Radiation dose, Signal-to-noise ratio

## Abstract

**Introduction:**

This study aimed to investigate the clinical feasibility of using an ultra-dilute, low-concentration iodine contrast agent in combination with dual-layer spectral computed tomography (CT) for pulmonary artery imaging.

**Methods:**

A total of 143 patients who underwent CT pulmonary angiography (CTPA) between May 2024 and May 2025 were enrolled. After excluding 19 patients with suboptimal image quality due to technical factors, 124 patients were included in the final analysis (spectral group: n = 64; control group: n = 60). Patients were assigned to either a spectral group (100 kV, 160 mgI/mL contrast, 25 mL, 2.5 mL/s) or a control group (120 kV, 350 mgI/mL contrast, 35 mL, 4 mL/s). In the spectral group, images were reconstructed using conventional CT and virtual monoenergetic imaging at 40, 70, and 110 keV. The control group underwent conventional CT imaging only. Image quality, CT attenuation values, signal-to-noise ratio (SNR), contrast-to-noise ratio (CNR), and radiation doses were assessed and compared between the two groups.

**Results:**

In the spectral group, 40 keV monoenergetic images yielded the highest CT values, SNR, and CNR for all pulmonary artery branches. The control group demonstrated superior image quality on conventional CT and at higher monoenergetic levels (70 and 110 keV), with statistically significant differences (*P* < 0.05). However, no significant differences were observed between the 40 keV spectral images and the control group. Subjective image quality scores were significantly lower at 70 and 110 keV, but comparable at 40 keV (*P* > 0.05).

**Discussion:**

The use of an ultra-low concentration iodine contrast agent (160 mgI/mL) combined with dual-layer spectral CT provides diagnostic image quality equivalent to that of conventional protocols, while reducing the iodine load by 67% (to 4 g per examination). This approach reduces iodine load while maintaining diagnostic image quality, although its effect on renal safety requires further clinical validation.

**Conclusion:**

Dual-layer spectral CT combined with 40 keV virtual monoenergetic imaging allowed pulmonary artery imaging with markedly reduced iodine load while maintaining diagnostic image quality comparable to that of the conventional protocol.

## 1 INTRODUCTION

Pulmonary embolism (PE) is a life-threatening condition caused by the obstruction of the pulmonary artery or its branches by a thrombus. It is the third most common cardiovascular cause of death after myocardial infarction and ischemic stroke [1]. Delayed or missed diagnosis of PE may lead to adverse clinical outcomes and increased mortality [2]. Therefore, a timely and evidence-based diagnostic evaluation is essential in patients with suspected PE [3]. Computed tomography pulmonary angiography (CTPA) is widely used as the preferred imaging modality for suspected PE because of its non-invasive nature, high spatial resolution, and rapid acquisition [4-7]. However, conventional CTPA protocols require high-concentration iodine contrast agents (270–370 mgI/mL), rapid injection rates (up to 5 mL/s), and relatively large volumes (60–100 mL); therefore, optimizing contrast administration remains clinically relevant, particularly in the context of iodine exposure, renal safety, and hypersensitivity-related concerns [8-10]. Recent advances in dual-layer spectral CT and related reconstruction techniques enable the generation of virtual monoenergetic images (MonoE), significantly enhancing vascular visualization [11-14]. This technology offers the potential to reduce contrast agent dosage while maintaining diagnostic image quality. However, optimizing contrast agent use without compromising diagnostic reliability remains a key challenge in current CTPA research. Low-energy monoenergetic imaging can increase the CT attenuation of iodine, thereby compensating for reduced contrast concentration. Therefore, we hypothesized that combining an ultra-dilute low-concentration iodine contrast agent (160 mgI/mL) with 40 keV virtual monoenergetic imaging using dual-layer spectral CT could substantially reduce iodine intake while preserving diagnostic image quality. This study aimed to validate the clinical feasibility of this protocol [15-18].

### 1.1 Contributions of the Study

- First application of an iodine contrast agent diluted to 160 mgI/mL (46% of conventional concentration) combined with dual-layer spectral CT for pulmonary artery imaging.
- Achievement of a 67% reduction in iodine intake (to 4 g per examination) through multi-parameter optimization, including low concentration, low injection rate, low tube voltage, and spectral reconstruction.
- Systematic comparison of image quality across different keV levels (40, 70, and 110 keV), identifying 40 keV as the optimal monoenergetic level.
- Demonstration that the ultra-low iodine protocol provides diagnostic image quality comparable to that of conventional CTPA.

## 2 MATERIALS

### 2.1 Patients

This was a prospective, single-center, controlled study designed to evaluate the feasibility of an ultra-low iodine contrast protocol in dual-layer spectral CT pulmonary angiography. Patients were assigned to either the spectral group or the control group based on the examination period: the spectral group included patients from May to November 2024, and the control group included patients from December 2024 to May 2025. All image acquisitions and analyses followed standardized operating procedures.

Inclusion criteria were: (1) clinically suspected PE; (2) no known allergy to iodinated contrast agents; and (3) signed informed consent. Exclusion criteria included: (1) confirmed hypersensitivity to iodinated contrast; (2) pregnancy or lactation; (3) altered mental status or severe anxiety precluding successful CTPA; (4) significant cardiac, hepatic, or renal dysfunction; and (5) poor image quality due to technical factors unrelated to the contrast protocol (*e.g*., motion artifacts, bolus tracking failure, equipment malfunction). Importantly, no cases were excluded solely due to low vascular enhancement, potentially related to the low-concentration contrast agent. After excluding 19 patients, 124 were included in the final analysis (spectral group: n = 64; control group: n = 60). The study flowchart is presented in Fig. (**1**).

This study was approved by the Ethics Committee of Civil Aviation General Hospital (Approval No. 2024-L-K-123) and conducted in accordance with the Declaration of Helsinki.

### 2.2 Contrast Injection and CT Acquisition

All examinations were performed using a dual-layer spectral CT scanner (Spectral CT 7500, Philips Healthcare, Netherlands). Patients were positioned supine with arms elevated, and scanned during free breathing from the thoracic inlet to the costophrenic angle. Automated bolus tracking was applied with a trigger threshold of 90 HU at the main pulmonary artery.

Scanning parameters were as follows: tube voltage of 100 kV for the spectral group and 120 kV for the control group; automatic tube current modulation (DoseRight) with a ReduceDose setting of 18; collimation of 128 × 0.625 mm; slice thickness and interval of 1 mm; pitch of 1.178; rotation time of 0.27 s; post-injection delay of 1.5 s; and post-threshold delay of 3.4 s.

For the spectral group, iopamidol (320 mgI/mL) was diluted 1:1 with saline to achieve a final concentration of 160 mgI/mL, and 25 mL was injected at 2.5 mL/s, followed by 25 mL saline at the same rate. The control group received undiluted iopamidol (350 mgI/mL) at 35 mL and 3.5 mL/s, followed by 30 mL saline at the same rate.

The parameter differences between groups were intentional: (1) lower tube voltage in the spectral group was used to enhance iodine contrast at low energy levels, compensating for reduced iodine concentration; (2) the spectral group protocol (low volume, low flow rate, diluted concentration) was designed to test the feasibility of ultra-low iodine load, while the control group followed standard clinical practice; (3) automatic tube current modulation ensured comparable image noise and radiation doses between groups.

### 2.3 Image Post-Processing

Raw data were transferred to a dedicated workstation (IntelliSpace Portal V9, Philips Healthcare) for post-processing. Spectral-based imaging (SBI) was used to generate virtual monoenergetic images at 40, 70, and 110 keV.

### 2.4 Image Quality Assessment

#### 2.4.1 Subjective Evaluation

Two experienced radiologists independently assessed image quality using a 5-point scale:

- 5: Excellent - clear vascular contours, good continuity, uniform density, no artifacts.
- 4: Good - generally clear contours, minor discontinuity in 1–2 segments, acceptable density uniformity, minimal artifacts.
- 3: Fair - somewhat unclear contours, discontinuity in 3–5 segments, acceptable density, visible artifacts.
- 2: Poor - poorly defined contours, discontinuity in >50% of segments, poor density uniformity, numerous artifacts.
- 1: Non-diagnostic - unclear contours, discontinuity in >60% of segments, poor density, severe artifacts.

#### 2.4.2 Objective Evaluation

CT attenuation values (in HU) and standard deviations (SD) were measured in the main, lobar, segmental, and subsegmental pulmonary arteries, as well as in the paravertebral muscles. SNR and CNR were calculated as follows:

- SNR = CT value / SD
- CNR = (CT value of artery − CT value of muscle) / SD

Radiation dose parameters, including CT dose index volume (CTDIvol), dose-length product (DLP), and effective dose (ED), were recorded. ED was calculated as DLP × k, with k = 0.014 mSv/(mGy·cm).

### 2.5 Statistical Analysis

Statistical analyses were performed using SPSS version 27.0 and GraphPad Prism version 10.0. Normality was assessed using the Kolmogorov–Smirnov test. Normally distributed data were expressed as mean ± SD; non-normally distributed data were presented as median with interquartile range. Group comparisons of demographic and radiation dose data were performed using the t-test or Mann–Whitney U test as appropriate. Inter-rater agreement was evaluated using the intraclass correlation coefficient (ICC). Subjective and objective image quality parameters were compared using ANOVA. A P-value < 0.05 was considered statistically significant.

## 3 RESULTS

### 3.1 Patient Characteristics

The median age was 65 years (range: 56–72 years), and the mean BMI was 24.39 ± 3.67 kg/m^2^. No significant differences were observed between the spectral group (n = 64) and control group (n = 60) in terms of sex, age, BMI, or radiation dose parameters (CTDIvol, DLP, ED; all *P* > 0.05; Table **1**).

### 3.2 Subjective Image Quality

Significant differences in subjective image quality were observed across energy levels in the spectral group (all *P* < 0.001). The 40 keV images received the highest scores (Rater A/B: 4.72 ± 0.60 / 4.67 ± 0.57), substantially higher than those at 70 keV (~2.27 ± 0.57) and 110 keV (~1.10 ± 0.52). Conventional CT images scored approximately 1.97 ± 0.76 between the 70 keV and 110 keV levels. No significant differences were found between the 40 keV spectral images and the control group (P = 0.12–0.57), indicating comparable clinical applicability. Inter-rater agreement was good for 70 keV (ICC = 0.834) and conventional CT (ICC = 0.835), and moderate for 40 keV (ICC = 0.610) and the control group (ICC = 0.657), suggesting more stable scoring at higher energy levels (Tables **2**-**4**).

### 3.3 Objective Image Quality

Significant differences in objective image quality were observed across energy levels in the spectral group (all *P* < 0.001). The 40 keV images demonstrated the highest SNR and CNR across all pulmonary artery branches, including the main, lobar, segmental, and subsegmental arteries. Notably, at the subsegmental level, the 40 keV group achieved a CNR of 19.86 ± 9.87 and an SNR of 21.81 ± 9.99, significantly outperforming other energy levels (*P* < 0.001), indicating a distinct advantage for visualizing distal small vessels.

**Fig. (2):**
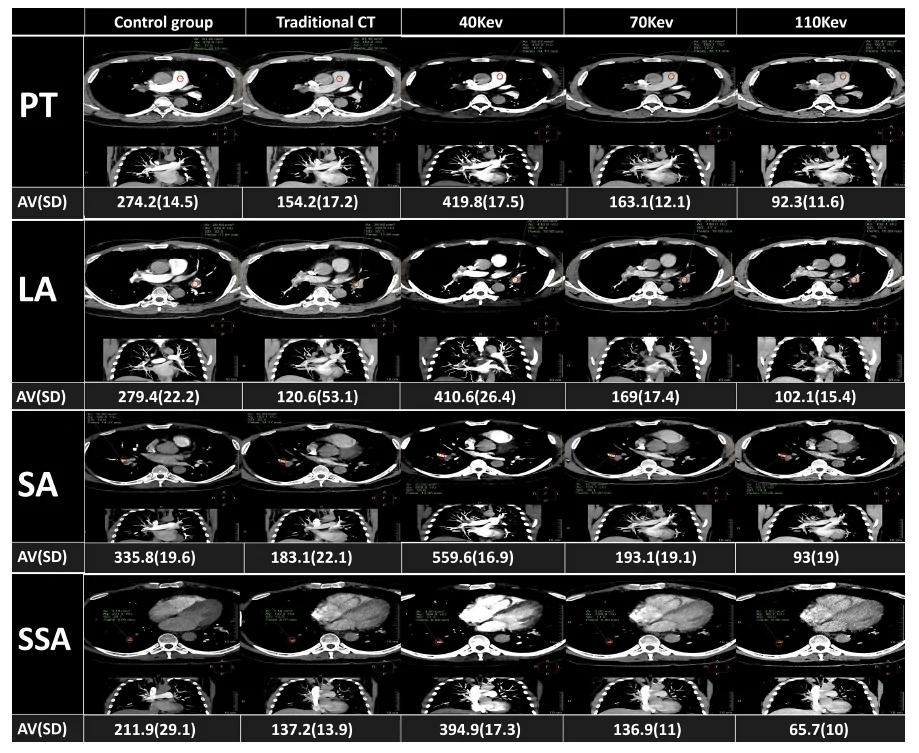
Shows the measurement methods of attenuation values (AV) and noise (SD) for the branches of the pulmonary artery using CT imaging at different energy levels. The left side represents the control group with traditional CT imaging, while the right side shows spectral imaging at 40 keV, 70 keV, and 110 keV, respectively. The ROIs (Region of interest) marked by red circles are located at the main trunk of the pulmonary artery, the lobar arteries, the segmental arteries, and the subsegmental arteries.

When comparing the 40 keV spectral group with the control group, no statistically significant differences were found in SNR or CNR for any pulmonary artery branch (P = 0.227–0.845), confirming that the ultra-low iodine protocol with 40 keV reconstruction yields objective image quality equivalent to that of conventional CTPA (Tables **5** and **6**; Fig. **2**). The distribution of SNR and CNR across imaging groups is shown in Figs. (**3** and **4**), respectively.

## 4 DISCUSSION

This study demonstrates that an ultra-low iodine CTPA protocol using 160 mgI/mL contrast agent (25 mL, 2.5 mL/s) combined with dual-layer spectral CT and 40 keV virtual monoenergetic imaging achieves diagnostic image quality comparable to that of conventional CTPA, while reducing the iodine load by 67% (to 4 g per examination). Because renal outcomes were not assessed in the present study, this finding should be interpreted as evidence of image-quality feasibility and iodine-load reduction rather than direct evidence of reduced contrast-associated kidney injury. This distinction is clinically relevant because acute kidney injury after CTPA in patients with acute pulmonary embolism has been reported and may be influenced by hemodynamic severity and right ventricular dysfunction [19].

The spectral group employed a lower tube voltage (100 kV *vs*. 120 kV), which enhances iodine contrast and improves CNR. Despite the voltage reduction, radiation doses (CTDIvol, DLP, ED) were comparable between groups, likely due to the adaptive tube current modulation (DoseRight), which maintained consistent image noise levels. Similar findings have been reported in coronary and CTA dual-low imaging studies [20-25].

Compared with previous research, the contrast concentration reduction in this study is more substantial. Kristiansen *et al*. [26] reduced contrast concentration to 240 mgI/mL, whereas we achieved 160 mgI/mL-a 33% further reduction. In terms of iodine intake, conventional CTPA protocols typically involve 12–15 g of iodine per examination. Through multi-parameter optimization, we reduced this to 4 g, which is 30–50% lower than the low-dose protocol reported by Lee *et al*. [27] (approximately 6–8 g). Moreover, unlike studies that focused solely on reducing contrast volume, we simultaneously optimized concentration, injection rate, tube voltage, and spectral reconstruction, demonstrating the advantage of a multi-dimensional approach [28-30].

The 40 keV monoenergetic images provided the highest vascular attenuation, effectively compensating for the reduced iodine concentration. Prior CTA studies have shown that lowering tube voltage from 120 kV to 100 kV can substantially increase iodine attenuation [31]. In our study, the combination of low tube voltage and 40 keV reconstruction yielded significantly improved CT values compared with conventional images, with no compromise in subjective or objective quality [32-35]. Notably, distal vessel enhancement was better preserved with low-concentration contrast, consistent with findings by Kristiansen *et al*. [26]. Recent pulmonary CTA and related contrast-enhanced CT studies have also shown that advanced detector technology, low-energy VMI, and reconstruction- or AI-based enhancement techniques can preserve or improve image quality while supporting dose or contrast-media reduction [36-39].

This study has several limitations. First, it was a single-center study with a relatively small sample size, which may introduce selection bias and limit statistical power, particularly for assessing rare adverse events. Stratified analysis by renal function was not performed, although patients with renal impairment may benefit most from low-iodine protocols. Second, we did not monitor post-scan renal function, as the primary aim was to evaluate image quality rather than clinical outcomes; therefore, the impact of low-concentration contrast on CIN incidence could not be assessed. Third, the generalizability of this protocol may be limited to institutions equipped with spectral CT, and standardized protocols across centers are needed. For obese patients (BMI > 30 kg/m^2^), parameter adjustments may be necessary to maintain image quality [28,40]. Finally, while Xue L *et al*. [29] reported no significant increase in mild adverse events in low-contrast spectral pulmonary artery CT angiography, larger studies are needed to determine whether combining low concentration with reduced flow rate further lowers adverse event rates.

## CONCLUSION

In conclusion, dual-layer spectral CT combined with 40 keV virtual monoenergetic imaging allowed CTPA to be performed with markedly reduced iodine load while maintaining subjective and objective image quality comparable to that of the conventional protocol. This protocol may be considered for selected patients when iodine dose reduction is desirable, but its impact on renal safety and broader clinical outcomes requires further prospective validation.

## Figures and Tables

**Fig. (1):**
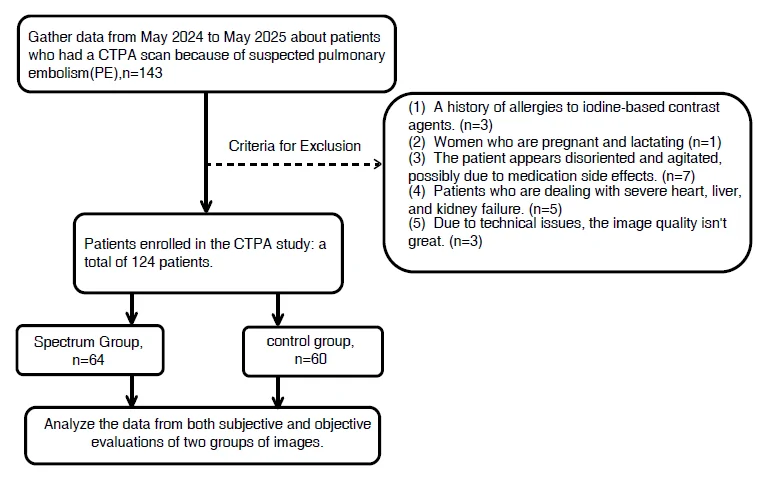
Flowchart for patient inclusion criteria.

**Fig. (3):**
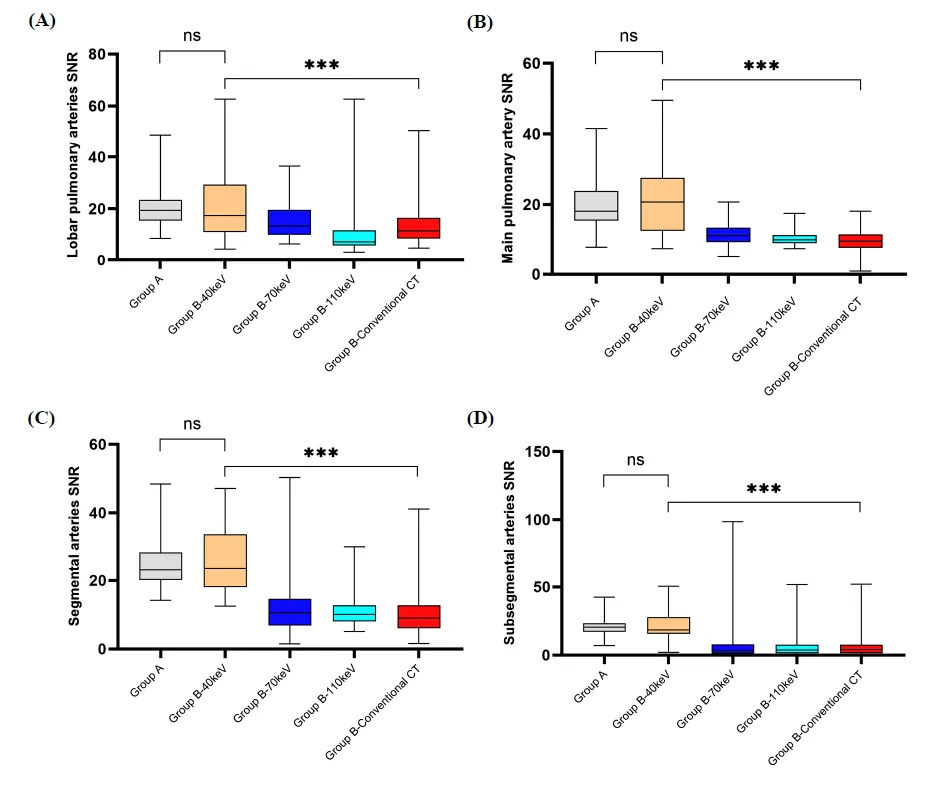
Box plots of the lobar pulmonary arteries SNR (**A**), main pulmonary artery SNR (**B**), segmental arteries SNR (**C**), and subsegmental arteries SNR (**D**) measured in the control group (conventional CT) and the spectral group at 40 keV, 70 keV, 110 keV, and conventional CT images. For SNR of pulmonary arteries at all levels, the 40 keV images yield significantly higher values compared to 70 keV and 110 keV images (*P* < 0.001), with no significant differences between the 40 keV spectral group and the control group. **Note: ** ***P < 0.001; ns = not significant (*P* > 0.05). The x-axis indicates the imaging groups, and the y-axis indicates the SNR values.

**Fig. (4):**
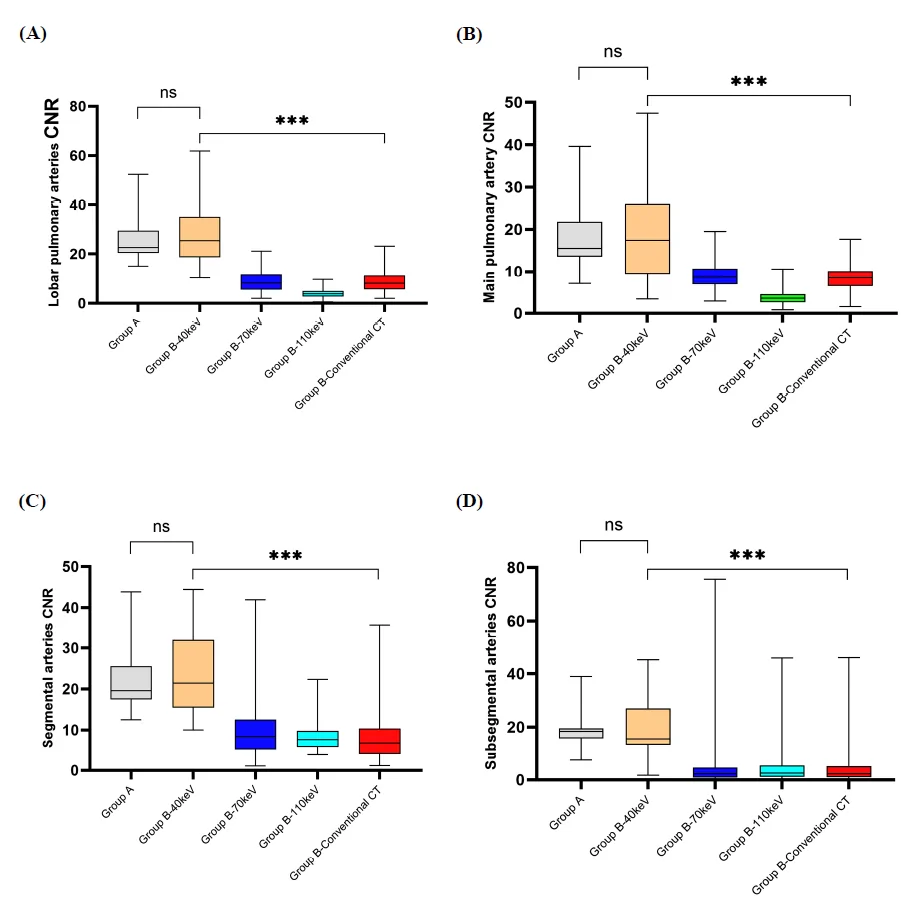
Box plots showing the lobar pulmonary arteries CNR (**A**), main pulmonary artery CNR (**B**), segmental arteries CNR (**C**), and subsegmental arteries CNR (**D**) in the control group (conventional CT images) and the spectral CT group at 40 keV, 70 keV, and 110 keV. For CNR of pulmonary arteries at all levels, the 40 keV images yield significantly higher values compared to 70 keV and 110 keV images (*P* < 0.001), with no significant differences between the 40 keV spectral group and the control group. **Note:** ***P < 0.001; ns = not significant (*P* > 0.05). The x-axis indicates the imaging groups, and the y-axis indicates the CNR values.

**Table 1:** Comparison of basic clinical information for two groups.

**Variable**	**Total (n=124)**	**Spectrum Group (n=64)**	**Control Group (n=60)**	**Statistical value**	***P***
Gender, n(%)	-	-	-	χ^2^=0.00	0.981
Women	58 (46, 77)	30 (46, 88)	28 (46, 67)	-	-
Man	66 (53, 23)	34 (53, 12)	32 (53, 33)	-	-
Age (year)	65(56-72)	62 ± 15	66(57-72)	U=1882	0.849
BMI (kg/m^2^)	24.39 ± 3.67	24.59 ± 3.47	24.17 ± 3.89	t=0.15	0.882
CTDIvol (mGy)	6.9(5.7-8)	7.39 ± 2.02	6.65(5.55-7.65)	U=1629.5	0.146
DLP (mGy·cm)	264(218.5-308)	277.81 ± 74.95	262(215-294)	U=1747.5	0.388
ED (mSv)	3.70(3.05-4.31)	3.89 ± 1.05	3.67(3.01-4.12)	U=1751.5	0.399

**Table 2:** Image quality scores for spectral groups.

Variable	Total(n=256)	40keV(n= 64)	70keV(n = 64)	110keV (n = 64)	Traditional CT images (n = 64)	Statistical value	*P*
Reviewer A, Mean ± SD	2.91 ± 1.56	4.72 ± 0.60	2.31 ± 0.56	1.09 ± 0.72	1.98 ± 0.72	F=502.95	<.001
Reviewer B, Mean ± SD	2.90±1.57	4.67±0.57	2.23±0.58	1.11±0.32	1.95±0.79	F=505.32	<.001

**Table 3:** Comparison of subjective image quality scores between the spectral group and the control group.

**Variable**	**Spectrum Group 40 keV(n = 64)**	**Control Group (n = 60)**	**Statistical value**	***P***
Reviewer A, Mean ± SD	4.72 ± 0.60	4.55 ± 0.59	t=1.57	0.12
Reviewer B, Mean ± SD	4.67 ± 0.57	4.62 ± 0.52	t=0.56	0.57

**Table 4:** Consistency analysis between raters.

**Group**		**ICC**	**95% confidence interval**		**Reliability level**
Spectrum	40keV	0.610	0.430	0.743	Medium
	70keV	0.834	0.739	0.896	Good
	110keV	0.746	0.614	0.838	Medium
	Conventional CT images	0.835	0.743	0.897	Good
Control		0.657	0.492	0.776	Medium

The ICC correlation coefficient less than 0.5 indicates poor consistency; 0.5 to 0.75 indicates moderate consistency; 0.75 to 0.9 indicates good consistency; greater than 0.9 indicates excellent consistency.

**Table 5:** Comparison of objective image quality of spectral groups.

Variable	Total (n = 256)	40keV (n = 64)	70keV(n = 64)	110keV(n = 64)	Conventional CT images (n = 64)	*P*
Main pulmonary artery SNR	13.21 ± 6.91	21.05 ± 9.05	11.6 ± 3.40	10.34±1.89	9.84 ± 3.35	<.001
Main pulmonary artery CNR	9.99 ± 7.54	18.46 ± 9.54	9.13 ± 3.19	3.74 ± 1.73	8.64 ± 3.33	<.001
Pulmonary artery leaf SNR	15.27 ± 10.22	20.79 ±12.43	15.4±7.05	11.23±10.5	13.64 ± 7.56	<.001
Pulmonary artery leaf CNR	12.3 ± 11.07	27.38 ± 11.54	8.87 ± 4.30	4.14 ± 2.03	8.89 ± 4.19	<.001
Pulmonary segmental artery SNR	14.79 ± 9.99	26.55 ±10.02	11.6± 7.43	10.8± 4.52	10.13 ± 6.29	<.001
Pulmonary segmental artery CNR	12.44 ± 9.77	24.52 ± 10.40	9.13 ± 6.18	8.16 ± 3.41	7.95 ± 5.47	<.001
Lobar segmental artery CNR	7.11 ± 11.55	19.86 ± 9.87	3.2 ± 10.20	2.71 ± 7.69	2.61 ± 7.66	<.001
Lobar segmental artery SNR	9.76 ± 11.90	21.81 ± 9.99	5.8 ± 12.70	5.68 ± 7.73	5.94 ± 7.69	<.001

Record the CNR and SNR values of the main pulmonary artery and branches in the comparative spectral group (40 keV, 70 keV, and 110 keV) and traditional CT images.

**Table 6:** Comparison of objective image quality between the spectral group and the control group.

**Variable**	**Total(n = 124)**	**Spectrum 40 keV(n=64)**	**Control(n=60)**	***P***
Main pulmonary artery SNR,	20.55 ± 7.89	21.05 ± 9.05	20.00 ± 6.47	0.457
Main pulmonary artery CNR	18.20 ± 8.23	18.46 ± 9.54	17.93 ± 6.62	0.719
Pulmonary artery leaf SNR	20.62 ± 10.26	20.79 ± 12.43	20.44 ± 7.37	0.845
Pulmonary artery leaf CNR	26.36 ± 9.77	27.38 ± 11.54	25.28 ± 7.38	0.227
Pulmonary segmental artery SNR	25.89 ± 8.96	26.55 ± 10.02	25.18 ± 7.69	0.395
Pulmonary segmental artery CNR	23.54 ± 9.19	24.52 ± 10.40	22.50 ± 7.64	0.219
Lobar segmental artery CNR	19.52 ± 7.95	19.86 ± 9.87	19.16 ± 5.24	0.619
Lobar segmental artery SNR	21.20 ± 8.22	21.81 ± 9.99	20.56 ± 5.85	0.396

## Data Availability

The data and supportive information is available within the article.

## LIST OF ABBREVIATIONS

AV: Attenuation value
BMI: Body mass index
CIN: Contrast-induced nephropathy
CNR: Contrast-to-noise ratio
CT: Computed tomography
CTDIvol: Volume CT dose index
CTPA: Computed tomography pulmonary angiography
DLP: Dose-length product
ED: Effective dose
HU: Hounsfield unit
ICC: Intraclass correlation coefficient
MonoE: Monoenergetic imaging
PE: Pulmonary embolism
ROI: Region of interest
SBI: Spectral-based imaging
SD: Standard deviation
SNR: Signal-to-noise ratio
VMI: Virtual monoenergetic imaging

## STANDARDS OF REPORTING

STROBE guidelines were followed.
